# Long-term prognosis of successful left ventricular substrate modification of electrical storm

**DOI:** 10.3389/fcvm.2022.981985

**Published:** 2022-08-31

**Authors:** Artak Margkarian, Harilaos Bogossian, Dirk Bandorski, Atisha Khan, Fuad Hasan, Tobias Fortmann, Klothilda Jahaj, Bernd Lemke, Markus Zarse

**Affiliations:** ^1^Helios Klinik Attendorn, Attendorn, Germany; ^2^Cardiology Department, Witten/Herdecke University, Witten, Germany; ^3^Evangelical Hospital Hagen-Haspe, Hagen, Germany; ^4^Faculty of Medicine, Semmelweis University, Budapest, Hungary; ^5^Klinikum Luedenscheid, Luedenscheid, Germany

**Keywords:** cardiac arrhythmia, electrical storm, ventricular tachycardia, catheter ablation, antiarrhythmic drugs

## Abstract

**Introduction:**

The Electrical storm (ES) subsumes a state of electrical instability of the heart, which manifests itself in repeated and potentially fatal ventricular arrhythmias (VA). We examine the long-term effects of substrate modification with regards to mortality, ventricular tachycardia (VT) recurrences and hospitalization depending on age, gender, heart function, scare location, VT documentation, postprocedural electrical stimulation (PES) and induced VTs.

**Methods:**

From 08/2008 and 09/2019 160 consecutive patients admitted for ES ablation *via* electroanatomical mapping were followed up until 04/2021.

**Results:**

50/160 patients showed VT recurrences after 13.8 ± 21.7 (0.0–80.3) months, with a characteristic steep curve directly after ES and then a rapid decline leading to a plateau (first month 10/50 (20%), first year 35/50 VT recurrences (70%) Mortality rates followed a similar pattern also the initial decline was not as steep. 42 patients died during the observation period (26%) after 16.6 ± 16.1 (0.0-67.9) months after ablation day (first month (*n* = 7, 16.7%) first year (*n* = 21, 50%). Gender, age, scare localization, missing VT documentation did not worsen outcome. Induction of >3 VTs and lack of PES due to hemodynamic instability significantly decreased effectiveness. Finding the entry significantly increased long-term effects.

**Conclusion:**

Ablation of ES is safe and feasible independent of a variety of factors. Employing more sophisticated tools to understand the reentry mechanism will further improve the long-term outcome.

## Introduction

The Electrical storm (ES) subsumes a state of electrical instability of the heart, which manifests itself in repeated and potentially fatal ventricular arrhythmias (VA) ranging from ventricular tachycardia (VT) to ventricular fibrillation (VF) (1, 2). It affects the sickest of the sick heart failure patients and was up to the 1990s associated with an extremely poor prognosis of mortality rates between 80 and 90% (3). The 2015 ESC guidelines, the 2017 AHA/ACC/HRS guidelines on the management of patients with VT and the 2019 HRS/EHRA/APHRS/LAHRS expert consensus statement on catheter ablation for VA define ES as >2 or ≥3 episodes of sustained VT/VF within 24 h (4–6). With the clinical introduction of implantable cardioverter-defibrillators (ICD), the mortality rate of ICD carriers 12 months after ES has decreased significantly to 33–54% (7). On the other hand we saw within the first 12–36 months after ICD implantation an incidence of ES in 10-25% of all ICD carriers (8, 9), with a more than five-fold increased mortality in the next 3 months compared with ICD carriers without ES. Patients with secondary prophylactic ICD implantation have a 10–40% risk, whereas patients with primary prophylactic implantation only have a 4% risk of suffering an ES in the first 12–26 months after implantation. Thus ICDs have not only changed prognosis and incidence of ES but also its definition and clinical appearance so that ES and ICD must be seen as two sides of the same coin. The most common mechanism of ES is a monomorphic VT maintained and sustained by a zone of slow conduction in the transition zone of a myocardial infarction, often many years ago. In the following paper we examine the long-term effects of left ventricular (LV) substrate modification by electroanatomical voltage mapping depending on a variety of cofactors.

## Methods

### Patient selection

The study included 179 patients who underwent substrate modification after ES between 08/2008 and 09/2019 and were followed up until 04/2021.

Five patients were excluded due to lack of endocardial substrate. Fourteen patients were lost to complete follow up, so that data from 160 patients could be retrospectively analyzed.

One hundred and fifty two patients were followed up during regular ICD visits. Eight patients who for various reasons did not receive an ICD were followed up by regular telephone visits by study nurse. The patients baseline characteristics are listed in Table 1.

**Table 1:** Characteristics of the patients at baseline.

**All Cases**	**160**
**Gender**	
Female sex—*n* (%)	21 (13,1)
**Age—year**	
Average	68.1
Median	69.2
standard deviation	±9.9
**Basic disease**	
Ischemic heart disease	118
Single vessel disease	26
Double vessel disease	41
Triple vessel disease	51
Coronary artery bypass surgery	41
Dilatative cardiomyopathy	38
Another cardiomyopathy's	4
**Ejection fraction**	
Average	30.6
Median	30.0
standard deviation	±10.6
Implantable cardioverter-defibrillator^a^	131
single chamber	46
Dual chamber	34
Three chamber	51
**Antiarrhythmic agents** ^a^	
Class I	14
Class II	136
Bisoprolol	28
Carvedilol	7
Metoprolol	94
Nebivolol	7
Class III	87
Other	9

a At the event.

### Substrate modification

In the majority of cases sedation was performed according to inhouse protocol with continuous intravenous administration of propofol and boli of midazolam and sufentanil. In the minority of patients general anesthesia including endotracheal intubation and mechanical ventilation was used. Two procedures were completed under mechanical circulatory support using Impella^®^ axial pump system. Systemic pressure was measured using invasive methods. Intraprocedural anticoagulation was performed using fractionated intravenous heparin administration based on activated clotting time (ACT) measurement with a target value of >300 s.

The LV was accessed by transmitral passage after echocardiography-assisted puncture of the intraatrial septum (*n* = 158) or transaortic access (*n* = 2). After basal conduction times were recorded, meticulous electroanatomic voltage mapping and three-dimensional reconstruction of the LV with identification of scar areas were performed using the Abbott NavX^®^ and Biosense Webster CARTO^®^ III mapping systems.

Areas of greatest scar location were divided into anterior, inferior, lateral and undetermined if there was a very diffuse scare distribution. Then, spontaneously occurring and provoked VTs were analyzed, late potentials were recorded and paced sites with long-stimulus QRS complexes marked.

Ablation end points were defined as ablation of exit or entrance points. Whenever possible, complex fragmented middiastolic or late potentials and paced long-stimulus QRS-complexes considered morphological substrate of the VT-entry were identified and ablated (*n* = 84). As an exit ablation strategy localized compact scars were ablated along the intermediate zone in between solid scar and healthy myocardium. As additional exit strategy, ablation lines from the scar edge to fixed anatomical obstacle such as the mitral valve ring were employed.

After completion of substrate modification we tried to re-induce the clinical VT by ventricular Stimulation with two basal cycle lengths and up to three extra-stimuli.

All peri- and postoperative complications were recorded.

### Study endpoints

To evaluate effectiveness of substrate modification we examined the following endpoints:

Primary endpoints: Death of any cause or recurrence of ES.

Secondary endpoints: Recurrence of VT with need for ICD Intervention like antitachycardia pacing (ATP) or shock and/or hospitalization. Hospitalizations were differentiated according to the main reason arrhythmias, cardiac decompensation, other cardiac reasons and noncardiac reasons, hospitalization due to cardiac decompensation (Hosp HF) > hospitalization for reason (Hosp cardiac) > hospitalization noncardiac (Hosp noncardiac). The observation period began immediately after ablation and continued until the occurrence of one of the primary end points. We further examined the effects of gender and age on VT outcome.

### Statistics

Statistical data were collected using a pseudonymized Microsoft^®^ Excel spreadsheet. Continuous variables were represented by mean, standard deviation, variance, and range. Graphs were generated using Microsoft^®^ Excel and PSPP statistical software Free Software Foundation, Inc. Hypothesis tests for two population proportions under the null hypothesis were determined using Z-test *via* “Social Science Statistics” calculator. Descriptive analysis using absolute and relative frequencies supported by odds ratio was performed using statistical and analysis software from “The R-Project for Statistical Computing”. Statistical significance was defined as *p*-values <0.05.

## Results

### Patient characteristics

We examined 160 patients (139 male, 21 female) with a mean age of 68.1 ± 9.9 years and a mean LV ejection fraction (EF) of 30.6 ± 10.6% for an observational period of 2.1 ± 1.9 (0–8,4) years. 118 showed coronary artery disease (CAD), respectively, ischemic cardiomyopathy (ICM), 38 dilatative cardiomyopathy (DCM). At time of admittance for Es ablation 133/160 patients wore ICDs (VVI *n* = 46, DDD *n* = 34, CRT *n* = 51) with a mean wearing time of 62.7 ± 47.7 (0.4–227.2) months. Nearly all patients had received antiarrhythmic (AA) drugs. Most of them 136/160 beta blocker (bisoprolol *n* = 28, carvedilol *n* = 7, metoprolol *n* = 94, nebivolol *n* = 7) and amiodarone (87/160) treatment. A minority of patients (14/160) received class I AA drugs (ajmaline *n* = 7, flecainide *n* = 1, lidocaine *n* = 5, mexiletine *n* = 1) or other AA or rhythm-acting drugs *n* = 9 (digitalis *n* = 4, procorolan *n* = 3, verapamil *n* = 2). Majority of patients received combination of two drugs, 9/160 patients received three of the above drugs.

### Complications

Twelve periprocedural complications (7.5%) were observed and treated accordingly. Most commonly we saw the occurrence of pericardial effusion (*n* = 6, 3.7%) requiring pericardiocentesis in 4 cases (2.5%). Other complications included transient ischemic attack (TIA) without any permanent neurological deficit (*n* = 1, 0.6%), tongue bite during endotracheal intubation (*n* = 1, 0.6%), puncture-related bleeding requiring intervention (*n* = 1, 0.6%) total AV block during ablation near the conduction system (*n* = 1, 0.6%) and moderate burn injuries at the site of the neutral electrode at the left lateral flank most likely due to increased stress triggered marked hyperhidrosis resulting in decreased attachment of the neutral electrode (*n* = 1, 0.6%).

### Ablation

Substrate modification was performed 6.3 ± 5.5 (0–30) days after ES, mostly due to delay until patients presented in the clinic with recurring ICD-shocks or referred from other clinics. In 20/160 patients immediate (*n* = 3) or ablation at the next morning (*n* = 17) were performed.

### Gender distribution

Most likely due to the low number of female patients with ES no differences in all examined study endpoints. There was a tendency for men to be more frequently admitted to the hospital for noncardiac reasons which is, however, not significant (Table 2).

**Table 2:** Analysis between male vs. female.

	**Male, *n =* 139**	**Female, *n =* 21**	***p*** **-value**	**OR**	**95% CI**
Exitus letalis	*n =* 35 (25.1%)	*n =* 7 (33.3%)	0.214	1.49	0.16–1.15
Recurrence of ES	*n =* 43 (30.9%)	*n =* 7 (33.3%)	0.413	1.12	0.31–2.19
VT NS	*n =* 25 (17.9%)	*n =* 3 (14.2%)	0.339	0.76	0.19–2.48
VT ATP	*n =* 21 (15.1%)	*n =* 3 (14.2%)	0.461	0.94	0.25–3.41
VT ICD shock	*n =* 30 (21.5%)	*n =* 2 (9.5%)	0.099	0.38	0.04–0.90
Hosp VT and ICD shock	*n =* 44 (31.6%)	*n =* 6 (28.5%)	0.388	0.86	0.28–2.14
Hosp HF	*n =* 29 (20.8%)	*n =* 4 (19.0%)	0.424	0.89	0.26–2.71
Hosp cardiac	*n =* 36 (25.8%)	*n =* 7 (33.3%)	0.237	1.43	0.18–1.27
Hosp noncardiac	*n =* 51 (36.6%)	*n =* 4 (19.0%)	0.056	0.41	0.04–0.35

### Age distribution

Table 3 depicts the differences between patients above and below 70 years of age. There were no differences observed in long-term outcome of arrhythmias or hospitalization. There is however increased mortality after VT ablation in the group ≥70 years of age (p=0.002).

**Table 3:** Analysis between age <70 vs. ≥70.

	**<70 a, *n =* 83**	**≥70 a, *n =* 73**	***p*** **-value**	**OR**	**95% CI**
Exitus letalis	*n =* 15 (17.2%)	*n =* 27 (36.9%)	**0.002**	**2.82**	0.00–0.01
Recurrence of ES	*n =* 27 (31.0%)	*n =* 23 (31.5%)	0.474	1.02	0.49–1.86
VT NS	*n =* 14 (16.0%)	*n =* 14 (19.1%)	0.304	1.24	0.27–1.38
VT ATP	*n =* 12 (13.7%)	*n =* 12 (16.4%)	0.320	1.23	0.27–1.53
VT ICD shock	*n =* 15 (17.2%)	*n =* 17 (23.3%)	0.170	1.46	0.16–0.74
Hosp VT and ICD shock	*n =* 26 (29.8%)	*n =* 24 (32.8%)	0.342	1.15	0.35–1.34
Hosp HF	*n =* 16 (18.3%)	*n =* 17 (23.2%)	0.223	1.35	0.21–0.96
Hosp cardiac	*n =* 22 (25.2%)	*n =* 21 (28.7%)	0.310	1.19	0.31–1.25
Hosp noncardiac	*n =* 29 (33.3%)	*n =* 26 (35.6%)	0.381	1.11	0.40–1.47

The bold values indicate the significant values.

### Cardiomyopathy and left ventricular ejection fraction

Table 4 compares the effects of substrate modification on DCM and ICM. The rare forms of cardiomyopathies like hypertrophic cardiomyopathy (HCM) and sarcoidosis (*n* = 4) were excluded from the analysis. DCM patients after substrate modification showed significantly more frequent VTs with consecutive ICD discharges (28.5 vs. 16.9%, *p* = 0.053) and significantly more frequent hospitalizations due to VT recurrence (*p* = 0.029). Table 5 depicts the effects of LV EF comparing patients with ES and a LV EF ≤ 30% and > 30%. Patients with poor LV EF displayed a significantly higher mortality (*p* = 0.008) but also significantly more frequent ICD shocks due to VTs (*p* = 0.028). Interestingly these patients also had significantly more frequent noncardiac hospital admissions (*p* = 0.037).

**Table 4:** analysis between ICM vs. DCM.

	**ICM, *n =* 118**	**DCM, *n =* 38**	***p*** **-value**	**OR**	**95% CI**
Exitus letalis	*n =* 33 (27.9%)	*n =* 9 (21.4%)	0.204	0.70	0.18–0.95
Recurrence of ES	*n =* 34 (28.8%)	*n =* 16 (38.0%)	0.133	1.52	0.13–0.56
VT NS	*n =* 20 (16.9%)	*n =* 8 (19.0%)	0.379	1.15	0.31–1.88
VT ATP	*n =* 15 (12.7%)	*n =* 9 (21.4%)	0.087	1.87	0.07–0.43
VT ICD shock	*n =* 20 (16.9%)	*n =* 12 (28.5%)	0.053	1.96	0.05–0.24
Hosp VT and ICD shock	*n =* 32 (27.1%)	*n =* 18 (42.8%)	**0.029**	**2.02**	0.03–0.12
Hosp HF	*n =* 34 (28.8%)	*n =* 9 (21.4%)	0.119	0.56	0.09–0.62
Hosp cardiac	*n =* 27 (22.8%)	*n =* 6 (14.2%)	0.177	0.67	0.15–0.82
Hosp noncardiac	*n =* 40 (33.8%)	*n =* 15 (35.7%)	0.416	1.08	0.40–1.74

The bold values indicate the significant values.

**Table 5:** Analysis between LV EF ≤ 30 vs. >30%.

	**≤30%, *n =* 83**	**>30%, *n =* 60**	***p*** **-value**	**OR**	**95% CI**
Exitus letalis	*n =* 29 (34.9%)	*n =* 10 (16.6%)	**0.008**	0.37	0.01–0.03
Recurrence of ES	*n =* 30 (36.1%)	*n =* 18 (30.0%)	0.221	0.76	0.22–0.90
VT NS	*n =* 16 (19.2%)	*n =* 9 (15.0%)	0.253	0.74	0.21–1.24
VT ATP	*n =* 13 (15.6%)	*n =* 9 (15.0%)	0.457	0.95	0.36–2.30
VT ICD shock	*n =* 22 (26.5%)	*n =* 8 (13.3%)	**0.028**	0.43	0.02–0.14
Hosp VT and ICD shock	*n =* 24 (28.9%)	*n =* 22 (36.6%)	0.164	1.42	0.16–0.66
Hosp HF	*n =* 16 (19.2%)	*n =* 12 (20.0%)	0.457	1.05	0.40–2.11
Hosp cardiac	*n =* 21 (25.3%)	*n =* 16 (26.6%)	0.427	1.07	0.40–1.82
Hosp noncardiac	*n =* 24 (28.9%)	*n =* 26 (43.3%)	**0.037**	1.88	0.04–0.15

The bold values indicate the significant values.

### Scar distribution

We differentiated between ICM and DCM. Whereas ICM showed a compact scar pattern all of the DCM patients showed a diffuse scare pattern. The scars were divided according to walls, corresponding to anterior (*n* = 54), inferior (*n* = 59), lateral (*n* = 23) and diffuse (DCM) (*n* = 22). Table 6 shows that independent of the scar location, substrate modification could be successfully performed. Patients with ES due to predominantly anterior scars were, however, more frequently hospitalized for cardiac decompensation (*p* = 0.019). Similar data is obtained when comparing the patients with anteriorly located scar with the patients with compact scar area of lateral and inferior localization (*p* = 0.022). Substrate modification in patients with diffusely distributed scars was less effective, showing significantly more hospitalizations for ICD shocks due to VTs (*p* = 0.021). There has to be taken into consideration that patients with diffusely distributed scars often overlapped with DCM patients (Tables 6, 7).

**Table 6:** Analysis between scar localization; anterior vs. the rest.

	**Anterior, *n =* 54**	**Rest, *n =* 106**	***p*** **-value**	**OR**	**95% CI**
Exitus letalis	*n =* 17 (31.4%)	*n =* 25 (23.5%)	0.142	0.67	0.14–0.59
Recurrence of ES	*n =* 17 (31.4%)	*n =* 33 (31.1%)	0.482	0.98	0.48–1.95
VT NS	*n =* 9 (16.6%)	*n =* 19 (17.9%)	0.422	1.09	0.35–2.01
VT ATP	*n =* 10 (18.5%)	*n =* 14 (13.2%)	0.187	0.67	0.15–0.91
VT ICD shock	*n =* 10 (18.5%)	*n =* 22 (20.7%)	0.369	1.15	0.32–1.70
Hosp VT and ICD shock	*n =* 15 (27.7%)	*n =* 35 (33.0%)	0.249	1.28	0.24–1.02
Hosp HF	*n =* 20 (37.0%)	*n =* 23 (21.6%)	**0.019**	0.47	0.02–0.08
Hosp cardiac	*n =* 13 (24.0%)	*n =* 20 (18.8%)	0.221	0.73	0.20–0.97
Hosp noncardiac	*n =* 19 (35.1%)	*n =* 36 (33.9%)	0.439	0.95	0.44–1.75

The bold values indicate the significant values.

**Table 7:** Analysis between scar localization; diffus vs. any localization compact.

	**Diffus, *n =* 22**	**Compact, *n =* 138**	***p*** **-value**	**OR**	**95% CI**
Exitus letalis	*n =* 4 (18.1%)	*n =* 38 (27.5%)	0.177	1.71	0.11–1.11
Recurrence of ES	*n =* 8 (36.3%)	*n =* 42 (30.4%)	0.289	0.77	0.23–1.48
VT NS	*n =* 6 (27.2%)	*n =* 22 (15.9%)	0.097	0.51	0.07–0.55
VT ATP	*n =* 5 (22.7%)	*n =* 19 (13.7%)	0.137	0.54	0.09–0.83
VT ICD shock	*n =* 7 (31.8%)	*n =* 25 (18.1%)	0.068	0.47	0.05–0.37
Hosp VT and ICD shock	*n =* 11 (50.0%)	*n =* 39 (28.2%)	**0.021**	0.39	0.02–0.10
Hosp HF	*n =* 3 (13.6%)	*n =* 30 (21.7%)	0.192	1.76	0.11–1.38
Hosp cardiac	*n =* 5 (22.7%)	*n =* 38 (27.5%)	0.318	1.29	0.22–1.85
Hosp noncardiac	*n =* 6 (27.2%)	*n =* 49 (35.5%)	0.225	1.47	0.17–1.22

The bold values indicate the significant values.

### VT documentation and postprocedural programmed electrical stimulation

Comparing patients with documented and not documented 12 lead electrocardiogram (ECG) clinical VT we saw no changes in any of the outcome parameters. Likewise, more than one documented clinical tachycardia did not worsen effectiveness of substrate morphology (Tables 8, 9).

**Table 8:** Analysis between no clinlincal VT vs. clinical VT available.

	**VT 0, *n =* 70**	**VT >0, *n =* 90**	***p*** **-value**	**OR**	**95% CI**
Exitus letalis	*n =* 15 (21.4%)	*n =* 27 (30.0%)	0.111	1.57	0.11–0.46
Recurrence of ES	*n =* 20 (28.5%)	*n =* 30 (33.3%)	0.260	1.25	0.26–1.02
VT NS	*n =* 16 (22.8%)	*n =* 12 (13.3%)	0.058	0.52	0.05–0.26
VT ATP	*n =* 11 (15.7%)	*n =* 13 (14.4%)	0.412	0.91	0.34–1.97
VT ICD shock	*n =* 16 (22.8%)	*n =* 16 (17.7%)	0.213	0.73	0.20–0.93
Hosp VT and ICD shock	*n =* 23 (32.8%)	*n =* 27 (30.0%)	0.349	0.88	0.36–1.37
Hosp HF	*n =* 14 (20.0%)	*n =* 19 (21.1%)	0.432	1.07	0.40–1.87
Hosp cardiac	*n =* 21 (30.0%)	*n =* 22 (24.4%)	0.216	0.75	0.21–0.87
Hosp noncardiac	*n =* 26 (37.1%)	*n =* 29 (32.2%)	0.258	0.80	0.27–0.99

**Table 9:** Analysis between one clinical VT vs. more than one clinical VT available.

	**VT 1, *n =* 65**	**VT >1, *n =* 25**	***p*** **-value**	**OR**	**95% CI**
Exitus letalis	*n =* 18 (27.6%)	*n =* 9 (36.0%)	0.221	1.47	0.17–1.18
Recurrence of ES	*n =* 24 (36.9%)	*n =* 6 (24.0%)	0.122	0.54	0.09–0.70
VT NS	*n =* 11 (16.9%)	*n =* 1 (4.0%)	0.053	0.20	0.01–0.87
VT ATP	*n =* 10 (15.3%)	*n =* 3 (12.0%)	0.341	0.75	0.17–2.72
VT ICD shock	*n =* 13 (20.0%)	*n =* 3 (12.0%)	0.187	0.55	0.10–1.44
Hosp VT and ICD shock	*n =* 20 (30.7%)	*n =* 7 (28.0%)	0.399	0.88	0.29–2.21
Hosp HF	*n =* 13 (20.0%)	*n =* 6 (24.0%)	0.339	1.26	0.23–2.04
Hosp cardiac	*n =* 16 (24.6%)	*n =* 6 (24.0%)	0.476	0.97	0.32–2.79
Hosp noncardiac	*n =* 24 (36.9%)	*n =* 5 (20.0%)	0.062	0.43	0.04–0.37

During substrate modification we induced VTs in 134 patients (1 VT in 38 patients, 2 VTs in 48 patients, 3 VTs in 30 patients, 4 VTs in 7 patients, 5 VTs in 6 patients and 6 VTs in 5 patients). Patientswith more than 3 VTs showed a significant increase in ES recurrence (41.6 vs. 21.4, *p* = 0.041). We refrained from postprocedural programmed electrical stimulation (PES) to avoid hemodynamic instability in 50 patients. These patients showed significantly more frequent ES (22/50 vs. 28/110, *p* = 0.009) and hospitalizations due to VT and ICD shocks (21/50 vs. 29/110, *p* = 0.024) during the observation period after substrate modification when compared to patients with postprocedural PES. In 19/110 cases we induced nonspecific, nonclinical VT or VF could be induced, which were not followed up. In all other cases no more tachycardias could be induced (Table 10).

**Table 10:** Analysis between postprocedural PVS vs. waiver or impossible PVS.

	**PVS, *n =* 110**	**no PVS, *n =* 50**	***p*** **-value**	**OR**	**95% CI**
Exitus letalis	*n =* 29 (26.3%)	*n =* 13 (26.0%)	0.481	0.98	0.45–2.06
Recurrence of ES	*n =* 28 (25.5%)	*n =* 22 (44.0%)	**0.009**	**2.30**	0.01–0.04
VT NS	*n =* 24 (21.8%)	*n =* 50 (12.0%)	0.109	0.55	0.03–0.26
VT ATP	*n =* 19 (17.3%)	*n =* 5 (10.0%)	0.116	0.53	0.08–0.66
VT ICD shock	*n =* 21 (19.0%)	*n =* 11 (22.0%)	0.335	1.20	0.29–1.52
Hosp VT and ICD shock	*n =* 29 (26.4%)	*n =* 21 (42.0%)	**0.024**	**2.02**	0.02–0.10
Hosp HF	*n =* 26 (23.6%)	*n =* 7 (14.0%)	0.081	0.53	0.07–0.40
Hosp cardiac	*n =* 30 (27.2%)	*n =* 13 (26.0%)	0.433	0.94	0.41–1.85
Hosp noncardiac	*n =* 39 (35.4%)	*n =* 16 (32.0%)	0.335	0.86	0.33–1.36

The bold values indicate the significant values.

### Ablation of entrance strategy

Late potentials were detectable in 60 patients and long-stimulus-QRS complexes at up to four areas in 61 subjects. Ablation in these areas improved ablation outcome significantly when compared to patients where these signs could not be detected (29/68 vs. 21/92, *p* = 0.004) (Table 11).

**Table 11:** Analysis between no entrance vs. present of entrance and ablation.

	**No entrance, *n =* 68**	**Entrance, *n =* 92**	***p*** **-value**	**OR**	**95% CI**
Exitus letalis	*n =* 22 (32.3%)	*n =* 20 (21.7%)	0.066	0.58	0.06–0.27
Recurrence of ES	*n =* 25 (36.7%)	*n =* 25 (27.1%)	0.098	0.64	0.10–0.38
VT NS	*n =* 13 (19.1%)	*n =* 15 (16.3%)	0.322	0.82	0.28–1.46
VT ATP	*n =* 12 (17.6%)	*n =* 12 (13.0%)	0.210	0.70	0.18–1.00
VT ICD shock	*n =* 17 (25.0%)	*n =* 15 (16.3%)	0.087	0.58	0.08–0.38
Hosp VT and ICD shock	*n =* 29 (42.6%)	*n =* 21 (22.8%)	**0.004**	**0.40**	**0.00–0.01**
Hosp HF	*n =* 16 (23.5%)	*n =* 17 (18.4%)	0.218	0.74	0.20–0.94
Hosp cardiac	*n =* 18 (26.4%)	*n =* 25 (27.1%)	0.460	1.04	0.45–1.87
Hosp noncardiac	*n =* 23 (33.8%)	*n =* 32 (34.7%)	0.450	1.04	0.46–1.74

The bold values indicate the significant values.

### VT recurrences and mortality

**Figure 1:**
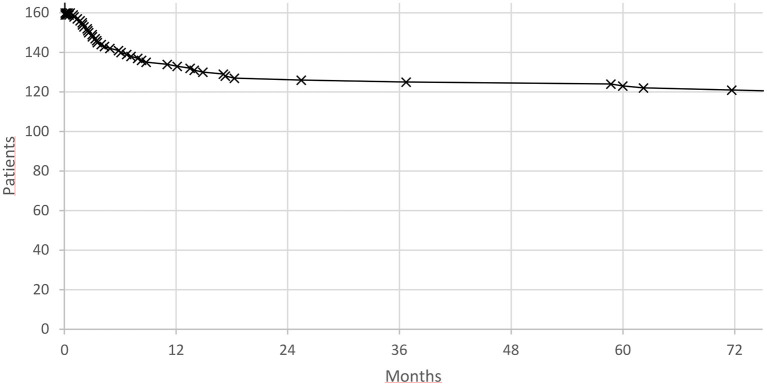
Electrical storm recurrence.

We noted 50/160 patients with VT recurrences on average after 13.8 ± 21.7 (0.0–80.3) months, with a characteristic steep curve at the beginning and then a rapid decline and plateau later in the course (Figure 1).

**Figure 2:**
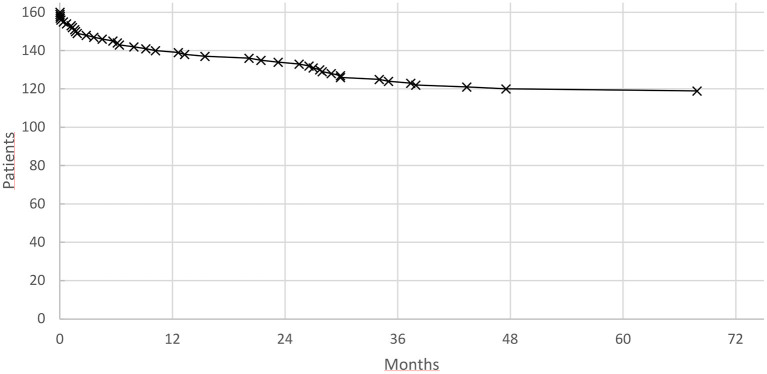
Mortality after substrate modification for electrical storm.

The event rate in first month was 10/50 (20%) and in the first year 35/50 VT recurrences (70%), respectively. Mortality rates followed a similar pattern also the initial decline was not as steep (Figure 2).

Forty two patients died during the observation period (26%) after 16.6 ± 16.1 (0.0–67.9) months after ablation day with one sixth within the first month (*n* = 7, 16.7%) and half of the patients within the first year (*n* = 21, 50%).

### Limitations of the study

This study was a retrospective analysis. Within the very long observation period modifications of ablation strategy and equipment have taken place which might have led to even better results. In this study we also exclusively used a first line solely endocardial approach even for patients with DCM with the concept that there might be transmural ablation of epicardial scar tissue possible. To calculate VT recurrences, we nearly exclusively relied on ICD interrogation possibly missing out on nonsustained very slow VTs.

## Discussion

In our study we could show that substrate modification of ES is safe and feasible. There is the greatest risk for recurrence within the first year. We saw a total of 42/160 patients (26.2%) dying during the observation period of up to 8.4 years averaging 2.1 ± 1.9 years with a 30-day mortality of 7/160 patients (4.4%) and one-year mortality of 21/160 patients (13.1%). Most investigations show a distinctly higher mortality rate during the observation period for patients with ES as compared to those without ES ranging from 22 vs. 2% (*p* < 0.001) (10) to 53 vs. 14% (p ≤ 0.001) (11). We also only saw 50/160 patients with VT recurrences (31%).

During postprocedural PES in our study, we could not induce the clinical VT, but we could induce nonclinical VTs in 19/110 cases (17%) and did not attempt for further ablation. This is in line with the largest studies that analyzed ES in patients with ICM where no clinical VTs could be induced in 60 to 80%, only nonclinical VTs could be induced in 15 and 25% and in 0–10% the clinical VT could still be induced (12).

In our study we reported 12 periprocedural complications (7.5%) most of them being minor and major complications being confined to pericardial effusion (*n* = 6, 3.7%) requiring pericardiocentesis in 4 cases (2.5%). This is in line with most studies reporting overall complication rates between 6.2 and 15% with major complication rates between 4.1 (13) and 3% (14), respectively.

We also investigated the effectiveness of our substrate modification with respect to gender age, scar localization, documented 12 lead ECG, induced VT and postprocedural PES, which appears to be the best evaluated end point of VT ablation, particularly in ICM patients associated with better arrhythmia-free survival (15).

The effectiveness of substrate modification in our study is independent of gender, although female patients are clearly in the minority. Concerning age, we could demonstrate that even very old patients clearly benefit from substrate modification. According to the rules of nature they die earlier than younger patients, but neither with respect to arrhythmia recurrences nor to hospitalizations they benefit less than younger patients. This is not as trivial as it seems as aging is a major risk factor for cardiac morbidity and mortality and cardiac aging impairs cardiac physiology leading to myocardial sarcopenia, hypertrophy, vascular hyperpermeability, inflammation and fibrosis (16).

Along these lines it is also understandable that patients with a more decreased EF die earlier and have more recurrent VTs. With respect to these findings, we examined the differences in scar locations, which plays an important role in determining function, remodeling, and prognosis following myocardial infarction (MI). MI on the anterior wall of the LV have long been known to lead to greater functional detriment and worse clinical outcomes than similarly sized MI in other locations. Several studies reporting clinical follow-up of patients suffering a first MI have found that anterior MI lead to greater risk of chronic heart failure (HF) and mortality (17, 18). In our study we confirmed these results showing much more hospitalizations for heart failure in patients with anterior infarction. Besides that, however, we did not elucidate any differences in effectiveness of dense endocardial scar ablation. In a subset of patients, however, we saw significantly less effects on ablation in suppressing VTs. These were patients with diffuse scars, synonymously for patients with DCM. Compared to ICM with very often localized dense endocardial scare areas with adjacent areas of slow conduction which were easily accessible to endocardial voltage mapping and ablation. In stark contrast the underlying substrate during DCM is much more complex most often sup-epicardial and intramural in location (19). In our study we choose a common diagnostic and therapeutic approach for all VT patients, hoping to also in DCM patients get a glimpse of the scar architecture by endocardial mapping and hoping to create transmural ablation lesions in the scar area thereby also affecting the more complex scares of DCM. Although an endocardial approach might still be an initial option it has to be further supplemented by an epicardial ablation for sufficient treatment of a DCM patient.

Oftentimes we stress to our students the importance of documentation of clinical VTs before ablation, which give hints into multiple directions from origin to differentiating between polymorphic vs. monomorphic VT and recognizing the underlying cardiac pathology and history. Of note we found it striking that although we did not have a 12 lead ECG documentation of the VT in 70 cases at the start of the procedure, effectiveness of the ablation in no way suffered. On the other hand, likelihood for recurrent ES and VTs dramatically increased with number of pre-ablation VT morphologies and decreased with noninducibility during PES after substrate modification, which is in line with previous studies. Still the future for ablation of ES is bright as we could show that the more, we understand the underlying mechanism allowing us for more precise intervention the better the effectiveness and the outcome in this still challenging and sometimes even daunting field of electrophysiology will be.

## Data availability statement

The original contributions presented in the study are included in the article/supplementary material, further inquiries can be directed to the corresponding author/s.

## Ethics statement

The studies involving human participants were reviewed and approved by Ethik-Kommission of Witten-Herdecke University. Written informed consent was not required due to the retrospective nature of the study.

## Author contributions

All authors listed have made a substantial, direct, and intellectual contribution to the work and approved it for publication.

## Conflict of interest

The authors declare that the research was conducted in the absence of any commercial or financial relationships that could be construed as a potential conflict of interest.

## Publisher's note

All claims expressed in this article are solely those of the authors and do not necessarily represent those of their affiliated organizations, or those of the publisher, the editors and the reviewers. Any product that may be evaluated in this article, or claim that may be made by its manufacturer, is not guaranteed or endorsed by the publisher.

## Abbreviations

AA: antiarrhythmic
ACT: activated clotting time
ATP: antitachycardia pacing
CAD: coronary artery disease
DCM: dilated cardiomyopathy
ECG: Electrocardiogram
EF: ejection fraction
ES: Electrical storm
HCM: hypertrophic cardiomyopathy
HF: heart failure
Hosp: hospitalization
ICD: ICD Shock
ICD: implantable cardioverter-defibrillators
ICM: ischemic cardiomyopathy
LV: left ventricular
MI: myocardial infarction
PES: postprocedural electrical stimulation
PES: programmed electrical stimulation
TIA: transient ischemic attack
VA: ventricular arrhythmias
VF: ventricular fibrillation
VT NS: nonsustained ventricular tachycardia
VT: ventricular tachycardia.
